# Which Plant Proteins Are Involved in Antiviral Defense? Review on In Vivo and In Vitro Activities of Selected Plant Proteins against Viruses

**DOI:** 10.3390/ijms18112300

**Published:** 2017-11-01

**Authors:** Oskar Musidlak, Robert Nawrot, Anna Goździcka-Józefiak

**Affiliations:** Department of Molecular Virology, Institute of Experimental Biology, Faculty of Biology, Adam Mickiewicz University in Poznań, 61-614 Poznań, Poland; rnawrot@amu.edu.pl (R.N.); agjozef@amu.edu.pl (A.G.-J.)

**Keywords:** plant defense, antiviral proteins, ribosome-inactivating proteins, RNA-binding proteins, pathogenesis-related proteins, dicer-like proteins, Argonaute proteins, plant virus, RNA silencing

## Abstract

Plants have evolved a variety of defense mechanisms to tackle virus attack. Endogenous plant proteins can function as virus suppressors. Different types of proteins mediate defense responses against plant viruses. Pathogenesis-related (PR) proteins are activated upon pathogen infections or in different stress situations and their production is one of many components in plant defense. Ribosome-inactivating proteins (RIPs) suppress translation by enzymatically damaging ribosomes and they have been found to have antiviral activity. RNA-binding proteins (RBPs) bind to target RNAs via specialized RNA-binding domain and can directly or indirectly function in plant defense system against RNA viruses. Proteins involved in silencing machinery, namely Dicer-like (DCL) proteins, Argonaute (AGO) proteins, and RNA-dependent RNA polymerases (RDRs) confer innate antiviral defense in plants as they are able to degrade foreign RNA of viral origin. This review aims to provide a comprehensive and up-to-date picture of plant proteins participating in antiviral defense. As a result we discuss proteins conferring plant antiviral resistance and their potential future applications in different fields of life including agriculture and medicine.

## 1. Introduction

**Figure 1:**
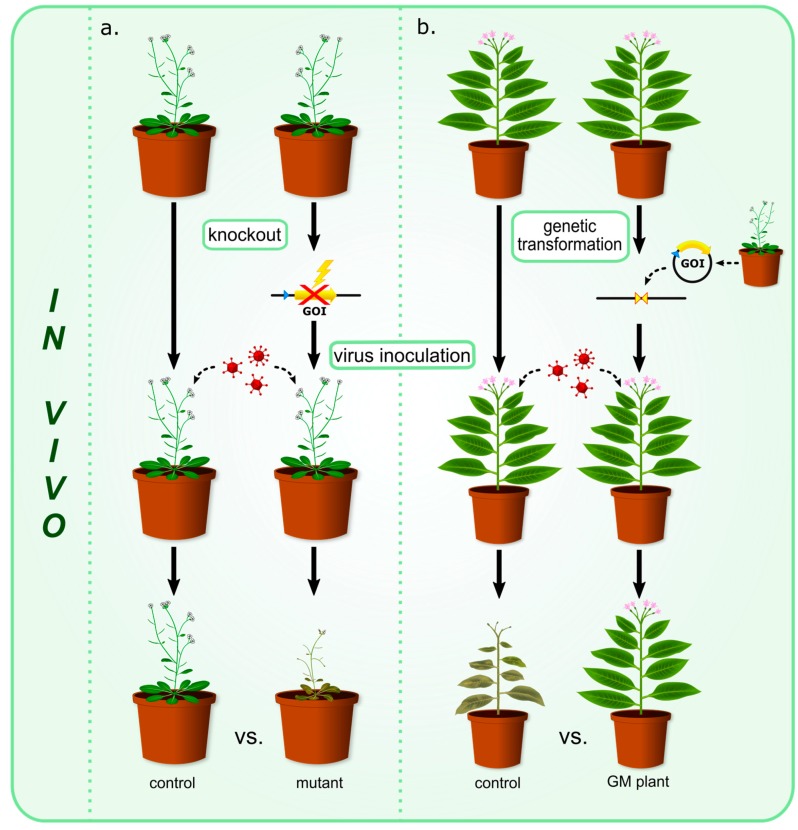

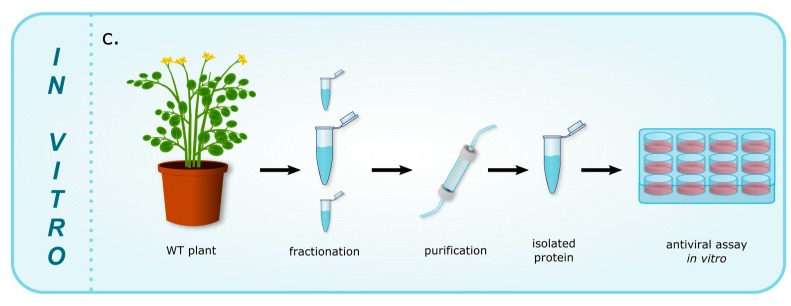
Schematic depiction of different strategies for studying antiviral protein properties. (**a**) Gene knockout technology employing loss-of-function mutant plants generated by performing genetic manipulations inactivating appropriate gene of interest (GOI); (**b**) genetically modified (GM) plants produced by the means of genetic transformation overexpressing particular protein of interest. After several days post-inoculation plants from both section (**a**,**b**) were compared with appropriate controls and analyzed using different bioassays (e.g., enzyme-linked immunosorbent assay (ELISA), quantitative polymerase chain reaction (qPCR), immunoblotting etc.); (**c**) isolation of a protein of interest using different fractionation and purification methods (e.g., ammonium sulfate precipitation, cation-exchange chromatography, etc.). Antiviral activity of isolated proteins was then assayed in vitro. In several studies more than one method for investigating protein antiviral activity was performed. The methods shown and described above are simplified for easier understanding of the main concept.

Plants possess a wide spectrum of defenses to deal with virus infections. They have inducible defense systems which are activated upon virus attack. The reaction to virus infection, among other elements, usually involves the production of different plant proteins which prevent further spreading of the virus. Pathogenesis-related (PR) proteins are activated in plants under pathological or similar circumstances, such as pathogen attack, wounding or exposure to particular plant hormones, salinity, cold, drought, etc. The production of PR proteins in the uninfected parts of affected plants can cease further virus infection. Antimicrobial peptides (AMPs) were found to have antiviral activities in vitro and they are considered as potential antiviral agents with possible biotechnological applications. Other plant proteins were also found to have antiviral properties in vitro. Such activity is characteristic for ribosome-inactivating proteins (RIPs), RNA-binding proteins (RBPs), and some defense-related proteins. Another way to tackle virus attack is RNA silencing which functions as a basic antiviral immune system in plants. Proteins playing a central role in the antiviral RNA silencing pathway include Argonautes (AGOs), RNA-dependent RNA polymerases (RDRs), and members of the plant Dicer-like (DCL) protein family. All these proteins are either presumed to be involved in defense responses of plants against viral infections or have been shown to possess antiviral activity in vitro which could be potentially utilized in agroeconomics. Nevertheless, mechanism of antiviral activity of plant proteins is still not fully understood. It is known that they can interact with viruses directly, occupy the virus infection site, inhibit the synthesis of virus protein and genome, or indirectly activate plant defense systems, thus enhancing resistance to viruses. In this review we have focused primarily on PR proteins, RIPs, RBPs, and proteins participating in RNA silencing as they are the main components of plant defense against viruses. The main premise behind writing this review was not only to summarize current knowledge regarding plant antiviral proteins but also to distinguish and discuss three classes of antiviral proteins—those which are a part of innate or induced defense response of plants to protect them against viral infection and those which are produced or cloned in other plants and exert antiviral effects against a wide range of plant and also animal viruses (Table 1). We distinguished three types of strategies commonly used for studying protein antiviral activity which are summarized in Figure 1. For some proteins antiviral activity was detected during in vivo studies involving gene knockout technology and mutant plants (Figure 1a). Another type of experiment used for antiviral in vivo activity assessment exploits transgenic plants or genetically modified plants overexpressing particular proteins (Figure 1b). The third case comprises proteins which were first isolated, and then their antiviral properties were studied using in vitro assays (Figure 1c). 

## 2. Plant Defense-Related Proteins

### 2.1. Plant Basal Resistance

Plants have evolved diverse defense mechanisms against pathogen infections. Complexity of plant immune system and efficiency to suppress pathogen infections differ between plant species [1]. Innate immunity, which is also called basal resistance, could be triggered by different features, like plant cell-wall derived components, which are released after pathogen attack or molecules presented by pathogens on the external side of the host cell. The latter comprise bacterial flagellins and lipopolysaccharides as well as fungal chitins and glucans and are called pathogen-associated molecular patterns (PAMPs) [2,3]. Plants from different families possess mostly conserved receptors recognizing PAMPs, called pattern recognition receptors (PRRs). Their stimulation leads to so-called PAMP-triggered immunity (PTI) [4]. On the other hand, plant pathogens (fungi, bacteria, viruses) carry different effectors and deliver them during infection to plant cells. In plant-pathogen “arms race” plants have evolved resistance (R) proteins, which can detect these effector molecules and induce defense responses. Such detected effectors are termed avirulence (Avr) proteins and pathogens carrying them are referred to as avirulent pathogens. These pathogens trigger plant defense, unlike virulent pathogens, which cause diseases [5]. Majority of Avr proteins are viewed as virulence factors that are necessary for the colonization of the host plant. When Avr proteins are recognized by resistant host plants, they act as “specific elicitors” of plant defense [6,7,8]. Most of the cloned *R*-gene products have leucine-rich repeat (LRR) domains coupled to a putative nucleotide binding site (NBS) domain. Recognition between corresponding plant R protein and pathogen Avr proteins induces effector-triggered immunity (ETI) (previously named gene-for-gene resistance) [1,5,7]. 

Defense responses induced by PTI or ETI are similar, however ETI is faster and accelerated and often leads to localized programmed cell death, which is referred to as hypersensitive response (HR) [1,2]. Within a few hours of HR, the plant starts to produce different PR proteins locally, at the infection site, and also systemically, throughout the whole plant [9]. Infection of the plant by many fungal, bacterial, and viral pathogens leads to systemic activation of a long-lasting and wide-ranging disease resistance termed systemic acquired resistance (SAR) [10]. SAR involves both local and systemic salicylic acid (SA) production and the activation of *PR* genes [11]. 

For plant viruses no conserved PAMP has been identified to date. The primary plant defense is based in this case mainly on RNA silencing [12]. However, PTI-based innate responses could contribute to antiviral plant defense as well. Recently plant antiviral immunity mechanism has been proposed as a global translation suppression caused by constitutive activation of NSP-interacting kinase 1 (NIK1) [13]. The protein is classified as PAMP recognition leucine-rich repeat receptor-like kinase (LRR-RLK) and was reported to be a virulence target of the *Begomovirus* (*Geminiviridae*) nuclear shuttle protein (NSP) [14]. It was shown, that constitutive activation of NIK1 leads to translocation of the downstream component RPL10 to the nucleus, where it interacts with LIMYB (L10-interacting MYB domain-containing protein), which results in translational suppression and enhanced tolerance to the virus [13]. This idea seems to be very interesting but needs further investigation to possibly extend it on broad plant–virus interactions.

### 2.2. Plant Pathogenesis-Related Proteins and Antimicrobial Peptides

Generally, the production of PR proteins and AMPs in plants in response to pathogen infections and to different stress situations is an important element of plant defense [15,16]. Accumulation of PR proteins in the remote uninfected plant parts induces SAR, preventing further development of the infection. PR proteins are encoded by the host plant and activated only in pathological or related circumstances [17]. PR genes are upregulated by different types of pathogens including viruses and also by the addition of chemical compounds that mimic the effect of pathogen infection or cause similar stress conditions. These substances can be plant hormones, including ethylene (ET), jasmonic acid (JA), and SA, as well as wound responses that activate the production of proteins, which also accumulate during infections [15,17]. PR proteins are organized into 17 families (numbered consecutively from PR-1–PR-17), mainly depending on their primary structure, but also serological and biological activities [18,19,20]. Several PR proteins are hydrolytic enzymes, e.g., chitinases and β-1,3-glucanases, which exhibit antimicrobial properties through the degradation of pathogen cell wall components. Some PR proteins appear to be antimicrobial agents because of their ability to bind to chitin, their structural similarity to osmotin or thionin, their proteinase-inhibiting activity and ribonuclease activity. Many of them have also direct or indirect antiviral properties (Table 1). Novel PR-9 protein possessing antiviral activity, was purified from *Stellaria media*. It was found that Stellarmedin A has a peroxidase activity and that it inhibits *Herpes simplex virus* type 2 (HSV-2) replication in vitro [21]. Ribonucleolytic activity of CaPR10, a PR-10 protein from *Capsicum annuum* was observed towards *Tobacco mosaic virus* (TMV) RNA, as well as against total pepper RNA or yeast tRNA (Table 1). It was shown that phosphorylation of this protein increases its ribonucleolytic activity to cleave viral RNAs [22]. Additionally, other PR proteins, namely PR2a and PR3 from *Nicotiana tabacum* were shown to have antiviral properties against TMV in vivo. [23]. 

PR-12 (defensins), PR-13 (thionins), and PR-14 (lipid transfer proteins) are also classified into the group of AMPs [20,21]. These are short sequence peptides which generally contain from 20 to 95 amino acid residues and are rich in cysteine [24,25]. Several families of AMPs have been described, including defensins, LTPs, hevein type peptides, knottin-type peptides as well as peptides isolated from the seeds of *Impatiens balsamina* (IB-AMPs). Some of them interact directly with the envelope of the virus, causing the permeation of the envelope and ultimately the lysis of the virus particle, while others can inhibit cell fusion or binding of the virus with human cells [24,26,27]. There are several purified plant AMPs with known antiviral activity against HIV in vitro [28,29] (Table 1). However, whether or not AMPs are truly involved in antiviral resistance in plants remains to be determined. 

## 3. Ribosome-Inactivating Proteins

RIPs are proteins ubiquitous in plant kingdom which can suppress translation by enzymatically damaging ribosomes [72]. Apart from the RNA *N*-glycosidase activity toward ribosomes, RIPs also possess a broad spectrum of additional enzymatic activities such as polynucleotide: adenosine and guanosine glycosidase activity. They are also presumed to have RNase and DNase activities [73,74,75,76]. First described RIPs were ricin and abrin, which are powerful toxins isolated from the seeds of *Ricinus communis* and *Abrus precatorius*, respectively [77,78,79]. History of the research on plant RIPs has been well summarized recently thus it is not the main focus of this review [80]. Many RIPs have been found in various plant species including Pokeweed antiviral protein (PAP) in *Phytolacca americana* [81], *Momordica* anti-HIV protein 30 kDa (MAP30) in *Momordica charantia*, *Gelonium* anti-HIV protein 31 kDa (GAP31) in *Gelonium multiflorum*, Cochinin B in seeds of *Momordica cochinchinensis* or saporin in *Saponaria officinalis* [82,83,84,85]. RIPs are expressed at various levels in different plants and the expression of some of them is enhanced upon infections and other stress situations, such as salinity and cold, after treatment with jasmonic acid or abscisic acid, and mechanical wounding [86,87,88,89,90,91]. 

### 3.1. Classification of RIPs

**Figure 2:**
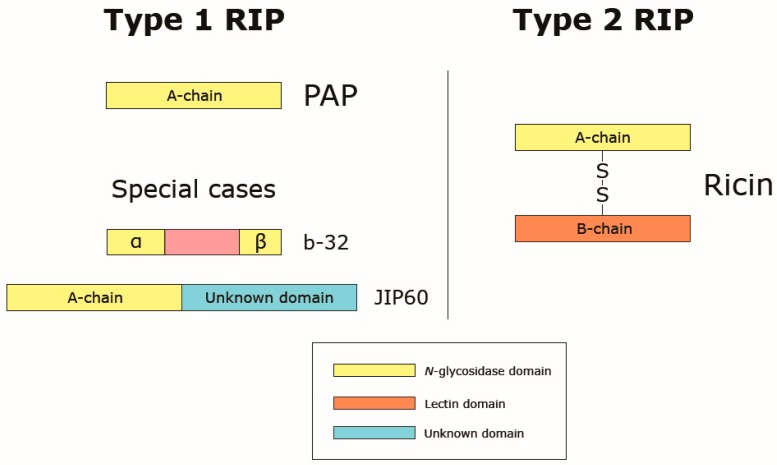
Schematic representation of the molecular structure of type 1 and type 2 RIPs. Special cases of type 1 RIPs refer to b-32 and JIP60 from maize and barley, respectively [98,99]. b-32 is synthesized in the form of proenzyme which becomes active only after the removal of a short internal peptide segment, leaving two segments of 16.5 and 8.5 kDa. JIP60 contains an active chain similar to classical type 1 RIP, which is linked to another segment of similar size but unknown function. The schematic representation of type 2 RIPs relates to mature ricin.

RIPs are categorized based on their structural properties into two major types, designated simply as type 1 and type 2. Type 1 RIPs are strongly basic monomeric enzymes consisting of a single peptide chain (A-chain) with *N*-glycosidase (EC 3.2.2.22) activity, and of approximately 30 kDa (Figure 2). Up-to-date the number of identified type 1 RIPs considerably exceeds type 2 RIPs [92,93]. Type 2 RIPs are composed of two peptide chains. The first chain, named A-chain, is similar to type 1 RIPs and it is linked to the second chain. The latter is referred to as B-chain of about 35 kDa and possesses lectin properties specific for sugars with galactose structure [94,95,96,97] (Figure 1). The B-chain interacts with galactosyl residues present on the surface of animal cells. Thereby it enables the A-chain to enter the cytoplasm, consequently leading to ribosome damage. This type of RIPs is much more toxic than type 1. Type 2 RIPs are heterodimeric (A–B) or tetrameric (A–B–B–A). The latter are four-chain proteins, composed of two dimers of the type (A–B) connected by a disulphide bridge.

Two proteins designated as b-32 and JIP60 isolated from maize and barley respectively, were found to be neither canonical type 1 nor type 2 RIPs what causes discrepancies regarding RIPs classification [98,99]. Some authors treat these proteins as completely different from the two other types and categorize them as type 3 RIPs. Type 3 comprises proteins consisting of a single A-chain with an additional peptide which must be removed for RIP’s activity [78,95,100]. Other authors classify only JIP60 as a type 3 RIP whilst b-32 is classified as type 1 [73,95]. However, to our best knowledge only two mentioned cases of such proteins have been described thus far. Therefore, it was suggested to consider these two proteins as special cases of type 1 RIPs (Figure 2) [93,101]. 

### 3.2. Antiviral Activity of RIPs

RIPs isolated from many different sources have been shown to efficiently inhibit both plant [60,102,103] and animal virus infections [104,105,106]. Several RIPs have been observed to inhibit viruses in vitro. Some others were found to confer plant immunity against viruses during in vivo studies. 

Transformation experiments involving RIPs were conducted to enhance the resistance of plants to plant viruses. PAP was found to increase resistance of tobacco and potato plants to infection of different viruses transmitted by aphids and mechanical damage, such as *Potato virus Y* (PVY), *Potato virus X* (PVX), and *Cucumber mosaic virus* (CMV) [60]. However, when PAP expression levels were high, toxic effect in tobacco and in bentgrass plants was observed [60,107]. Similarly, trichosanthin conferred resistance to transgenic tobacco against *Turnip mosaic virus* (TuMV) [65]. Potato plants transformed with *Phytolacca insularis* antiviral protein (PIP) were resistant against infection of PVY, PVX, and *Potato leafroll virus* (PLRV) [63]. Nonetheless, because of their toxicity all these RIPs generated a severe phenotype in transgenic plants. This led to several attempts where RIPs with lower toxicity toward host plants were used, including PAP II, which is a low-toxic isoform of PAP isolated from leaves [61]. It was also observed that PAP-C, a PAP mutant with removed C-terminus did not induce a detrimental effect in transgenic tobacco plants, where rRNA depurination was not observed [62]. Furthermore, PAP-C transformed tobacco plants are protected against PVX infection [62]. Because the ribosome inactivation is not imperative for antiviral activity of PAP-C, healthy tobacco lines exhibiting virus resistance could be obtained [62]. The plants transformed with RIP genes or with enhanced endogenous RIP levels showed increased resistance to viruses. This suggests that RIPs are significant players in plant adaptation and defense against pathogens and environmental stress conditions. 

Although, the evidence for antiviral activity of RIPs is compelling and the antiviral properties has been described for many type 1 and type 2 RIPs, the mechanism of antiviral activity has not been elucidated. Three ways of RIPs action have been suggested [73].

Some authors propose that thanks to the polynucleotide: adenosine glycosidase activity RIPs can act directly on virus particles and viral or virus-induced nucleic acids. This assumption came from the observation that RIPs depurinate nucleic acids [108]. However, it is presumed that RIPs do not directly affect intact viral particles, as treatment with PAP did not have any effect on TMV infectivity [109]. On the other hand, RIPs are typically localized in the intracellular space (e.g., saporins), in the protein bodies (e.g., ricin), in the cell wall matrix or in vacuoles (e.g., PAP), what segregates them from the cytosol where they could damage ribosomes and cease protein synthesis. In the so-called ‘local suicide’ model, when cells are damaged by virus vector (e.g., aphid) or by mechanical inoculation, and the integrity of the plasmalemma is disrupted, RIPs can enter the cytosol of infected cells where they can reach and inactivate protein synthesis machinery thus, killing infected cells, preventing viral replication and infection of neighboring cells [97]. Finally, it was proposed that RIPs can indirectly activate plant defense systems, thus inducing increased resistance to viruses.

However, experimental data often do not comply with the aforementioned potential ways of action. As outlined above, the ribosome inactivation is not necessary for PAP-C antiviral activity. Presumably, the antiviral activity of RIPs depends on an indirect activation of the plant defense system. Although evidence of such a mechanism has been obtained for recombinant RIPs expressed in transgenic tobacco, there is no data supporting this hypothesis in plants normally expressing RIPs [62]. 

Even though a specific biological function of RIPs still remains to be determined, it has been proposed that RIPs can be storage proteins, can participate in plant defense against pathogens and adaptation to environmental stress, and play a role in plant senescence [72,94,110]. 

## 4. RNA-Binding Proteins

Plants contain many RBPs, that are implied to interact with specific target mRNAs and change plant physiology. RBPs are diverse heterogenic proteins which participate in post-transcriptional regulation by direct association with RNA molecules. To bind target RNAs they utilize an RNA-binding domain (RBD) which can be sequence-specific or non-specific. These proteins play a critical role in cellular posttranscriptional gene regulation, polyadenylation, mRNA stability, RNA trafficking. They also participate in inhibition of RNA virus replication, movement, and translation. Therefore, RBPs may directly or indirectly participate in viral RNA-targeted defense system against RNA viruses at the transcriptional and translational level. For example, PR10 proteins including CaPR10, have RNase activity and are able to cleave viral RNA [58]. Other RBPs such as *Arabidopsis* Pumilio RNA-binding protein 5 (APUM5), associate directly with viral RNA and repress its translation disturbing viral replication and movement [58]. However, the molecular mechanisms of RBP-mediated defense against different pathogens are not fully understood. Some RBPs were reported to inhibit viral replication in vitro and several in vivo (Table 1). 

### 4.1. Structure of RBPs

RBPs comprise diverse modular structures, containing numerous repeats of few conserved domains, organized in variety of ways [111]. To be classified as an RBP a protein must contain at least one RBD. RNA Recognition Motif (RRM) and K Homology domain (KH) are the most common domains present in plant RBPs [112]. Other popular RBP domains are zinc finger domain (ZnF) [112,113], DEAD/DEAH box [114], cold-shock domain, Pumilio/FBF (PUF) domain [115], and Piwi/Argonaute/Zwille (PAZ) domain [116]. One RBP can have multiple binding domains which are often surrounded by auxiliary motifs, such as glycine-rich, arginine-rich, serine–arginine (SR) or arginine–glycine (RGG) [113,114,115,117,118]. 

### 4.2. Plant RBPs—In Vitro and In Vivo Activity against Viruses

RBPs from plants participate in immune response against viruses by direct and indirect interactions with viral RNA. Some RBPs associate with host RNA at mRNA levels and govern signaling pathways in a defense response to pathogens. Others can directly bind viral RNA and effectively affect its replication and movement. Hereafter, several examples of RBPs with antiviral activity are described (Table 1). 

APUM5 contains a conserved Pumilio homology domain (PHD) which is presumably responsible for the recognition and binding affinity of target RNAs [119]. PHD in APUM5 suppresses CMV replication by direct association with the “UGUA”-containing nucleotides in the 3’ UTR and with virus internal regions [58]. Furthermore, in one study, transgenic plants overexpressing APUM5 and wild-type plants were subjected to TuMV infection. At the initial stage, the transgenic plants displayed decreased RNA levels and marginally enhanced immunity in contrast to a wild-type plant [120]. Additionally, it is likely that APUM5 can govern unidentified host target RNAs in RNA sequence-specific manner [58].

*Arabidopsis thaliana* glycine-rich RNA-binding protein 7 (AtGRP7) participates in plant defense response against TMV and some bacterial pathogens [59]. In one study, after comparison of data from global transcript profiling between the wild-type plants and plants overexpressing AtGRP7, it was observed that AtGRP7 controlled about 300 transcripts, such as those responsible for circadian clock, ribosome function, RNA metabolism, and those participating in stress response [121]. 

As outlined above CaPR10 isolated from hot pepper (*C. annuum*) belongs to the pathogenesis-related protein family 10 (PR-10), which is characterized by ribonuclease-like activities. Because of its RNA-binding domain it is also classified as an RBP. CaPR10 is involved in plant defense by direct interaction and increased ribonucleolytic activity towards viral RNAs during viral infection [22]. Even though PR-10 proteins function as RBPs, their specific contribution to plant defense against RNA viruses remains to be determined since this protein family is involved in defense responses during various biotic and abiotic stress conditions [122,123]. The activities of the PR10 protein family are presumably non-specific, thus it was proposed that members of this family employ helper proteins to bind specifically to target RNAs of both viral and host origin. 

Two proteins belonging to *Arabidopsis* dsRNA-binding protein family were also found to exhibit antiviral activity in vivo. dsRNA-binding protein 3 (DRB3) and dsRNA-binding protein 4 (DRB4) among other classes of proteins such as DCLs, AGOs, and RDRs form RNA silencing machinery which enhances innate antiviral defense. *Arabidopsis* dsRNA-binding protein 3 (DRB3) physically associates with the RNA-directed DNA methylation (RdDM) pathway components including Dicer-like 3 (DCL3) and Argonaute 4 (AGO4) in the nucleus. RdDM pathway is used in plants to methylate viral genome as an epigenetic defense mechanism against geminiviruses [45]. Methylation of chromatin suppresses virus replication and transcription, and methylation deficiency results in plant hypersensitivity to geminiviruses. It has been demonstrated that DRB3, together with DCL3 and AGO4, participate in methylation-mediated antiviral defense [45]. Experiments were performed using two distinct types of geminiviruses, namely *Cabbage leaf curl virus* (CaLCuV) and *Beet curly top virus* (BCTV). From a panel of *Arabidopsis* drb mutants (drb2, drb3, drb4, drb5), only drb3 mutants were hypersusceptible to geminivirus infection, which correlated with a defect in the methylation pathway [45].

*Arabidopsis* dsRNA-binding protein 4 (DRB4) is involved in plant defense against the infection of TYMV [53]. DRB4 interacts with dicer-like 4 protein (DCL4) and both proteins are required for the formation of TYMV-derived small RNAs [52]. During virus infection DRB4 changes subcellular localization moving from the nucleus to the cytoplasm where it specifically interacts with viral RNA to control the infection [53]. DRB4 is also implied to regulate HR against *Turnip crinkle virus* (TCV) [54]. Moreover, DRB4 associates with tRNA-like structure (TLS), which is important for the replication and translation of viral RNA. DRB4 presumably functions as a translational repressor of RNA viruses in plants since it inhibits viral RNA translation, but not degradation. Nonetheless, whether DRB4 controls target host RNA and viral RNA at RNA or protein level, remains to be verified [58]. 

Recent results of Barton and colleagues show co-localizations of DRB2, DRB3, and DRB5 proteins with viral replication complexes (VRCs) in virus-infected plants [124]. Fluorescently tagged DRB2, DRB3, and DRB5 fusion proteins formed discrete concentrations in VRCs in *Arabidopsis* cells infected with *Tomato spotted wilt virus* (TSWV), TuMV, CMV, TYMV. Such observation implied probable interaction between these proteins and viral elements. Similarly, when *N. benthamiana* plants transiently expressing *Arabidopsis thaliana* dsRNA-binding proteins (AtDRBs) were infected with TuMV and TMV, the same three proteins (AtDRB2, AtDRB3, AtDRB5) accumulated within VRCs. Furthermore, in *N. benthamiana* plants expressing PVX-CFP fusion protein, AtDRB2, AtDRB3, AtDRB5 relocated from nuclei to cytoplasm, to eventually localize within VRCs. In another experiment, transgenic *Arabidopsis* plants with inactive DRB3 or DRB4, and wild-type plants were challenged with TuMV. After three weeks the virus titers increased in DRB3- and DRB4-defective plants [124]. These results demonstrate that DRB3 and DRB4 inhibit viral replication.

Protein binding to ToMV RNA 1 (BTR1) from *A. thaliana* interacts with the 5’ terminal region of *Tomato mosaic virus* (ToMV) and perturbs virus multiplication and local spreading [50]. The overexpression of BTR1 in *A. thaliana* leaves specifically inhibited ToMV infection, while the opposite effect was observed for BTR1 knockout plants [50]. The mechanism of BTR1 antiviral activity is not well understood. Presumably, BTR1 may contain specific binding domains for plant viral RNA, or it may be indirectly involved in host innate immunity. 

In general, little is known about the RBPs-mediated defense and its mechanism of action. It has been proposed that during pathogen infections RBPs control defense signaling genes at post-transcriptional and post-translational levels [58]. The knowledge about the mechanism of the effective virus RNA suppression via RBPs can be potentially exploited in transgenic crops as a synthetic virus defense strategy.

## 5. Proteins Involved in Innate Antiviral Defense via RNA Silencing Pathway

Plant genomes encode multiple proteins which participate in endogenous and foreign RNA silencing thus contributing to antiviral defense. These proteins include DCLs, AGOs or AGO-containing RNA-induced silencing complexes (RISCs), and cellular RDRs [34] (Table 1). RNA silencing is a basic antiviral defense mechanism which reinforces plant immune system. It operates to decrease viral RNA levels in infected cells and to block further spreading of the virus. Hereafter, a few classes of proteins involved in innate antiviral silencing are described.

### 5.1. Dicer-Like Ribonucleases

RNA silencing is triggered by Dicer-like ribonucleases III, which specifically cleave dsRNA or hairpin dsRNA regions of single-stranded RNAs (ssRNAs). The effects of cleavage are 21-26-nucleotide small interfering RNAs (siRNAs) or 21-22-nucleotide microRNAs (miRNAs) depending on their biogenesis and precursor structure [34,125,126,127]. Thus, siRNA is derived from perfectly paired dsRNA precursors and miRNA originates from imperfectly base-paired hairpin loop structures of the transcript of miRNA genes produced by RNA polymerase II. In general, different forms of viral RNA exist, which can act as viral small RNA (sRNA) precursors. Such precursors comprise double-stranded viral replicative intermediate RNAs (vRI-dsRNAs) of RNA viruses, as well as highly structured hairpin regions in viral ssRNA, and also mRNA of RNA and DNA viruses after processing into viral sRNAs. All small RNAs have a typical terminal region containing a 5’-phosphate group and two-nucleotide overhang at the 3’ end and they participate in sequence-specific RNA degradation, translation, inhibition and/or heterochromatin formation. 

RNA III ribonucleases are large multi-domain proteins which contain six types of domains: DEAD box (a conserved box in protein family of RNA helicases involved in ATPase activity), helicase C, DUF 283 (a conserved domain of unidentified function), PAZ (Piwi/Argonaute/Zwille), RNase III domain, and dsRBD (a conserved double stranded RNA binding domain) [128]. The PAZ, RNase III, and dsRBD domains participate in dsRNA binding and cleavage [129]. DCLs are essential elements in triggering antiviral response as they convert viral nucleic acids into siRNAs. Different classes of DCLs were described, like class I RNases, which have been identified in bacteria and yeast where they participate in the processing of cellular and viral RNA or classes 2 and 3, which play a key role in biogenesis of miRNA and/or siRNA in fungi, plants, invertebrate, and vertebrate. Additionally, the majority of class 3 Dicer RNases contain an N-terminal RNA helicase domain, which is closely related to RIG-like receptors (RLRs) and a PAZ domain, shared between Dicer and AGO proteins. The PAZ domain functions as RNA-binding domain which selectively recognizes the 2-nucleotide 3’-overhang of the siRNA duplex [130]. DCLs are ubiquitous in plants but their expression depends on various developmental stages as well as on biotic and abiotic stresses. Different DCLs participate in various ways in the biogenesis of distinct classes of endogenous small RNAs and in immunity [125]. As an example, four different DCLs have been identified in *A. thaliana* [51]. DCL1 from *A. thaliana* mainly participates in the production of miRNAs triggered by biotic and abiotic stresses. DCL2 produces siRNAs from natural cis-acting antisense transcripts and participates in viral resistance. DCL3 generates 24-nucleotide long repeat-associated siRNAs (rasiRNAs) that guide chromatin modification. DCL4 produces 21-nucleotide long siRNAs which act during post-transcriptional silencing [127]. Generally, plants attacked by DNA viruses of family *Caulimoviridae* and *Geminiviridae* generate 21, 22, and 24-nucleotide long virus-derived small interfering RNAs (vsiRNAs) which are produced by all four DCLs (DCL1, 2, 3, 4), while plants infected by RNA viruses accumulate vsiRNAs produced only by DCL4 and DCL2 [12]. DCL4 and DCL2 together with DCL4 were found to exert antiviral activity against various viruses infecting *A. thaliana* including PVX, TuMV, BMV, and TCV [30,33,34,51]. In *Nicotiana benthamiana* mutant plants DCL4 was shown to function against PVX [51]. 

### 5.2. RNA-Dependent RNA Polymerases

Apart from DCLs, siRNA synthesis in plants is additionally performed by RDRs. RDRs were the first RNA silencing elements to be discovered. They contain a conserved RNA-dependent RNA polymerase catalytic domain. RDRs were identified in RNA viruses, plants, fungi, Protista, in some animals but are absent in *Drosophila*, mice, and humans [131,132]. In plants, endogenous RDRs convert ssRNA into dsRNA, which is processed by Dicer-like nucleases into siRNA. RDRs can synthesize siRNA-producing dsRNA in a primer independent or primer dependent manner using a small RNA as a primer. These enzymes participate in siRNA biogenesis, in the production of vsiRNA, and in innate antiviral defense. In the genome of *A. thaliana* six RDR genes encoding six RDR proteins were identified. RDR1 is involved in the production of viral siRNAs from positive-strand RNA viruses, RDR2 participates in the synthesis of the DCL3-dependent 24-nucleotide rasiRNA and plays a role in sequence-specific heterochromatic silencing. RDR6 is responsible for the production of many classes of siRNAs such as these derived from transgene transcript [132,133,134]. Mainly RDR1 and RDR6 from *A. thaliana* were reported to have antiviral properties in vivo towards different plant viruses including TuMV, TCV, and BMV [30,34,54]. In another study transgenic tobacco (*N. tabacum*) plants with reduced numbers of RDR1 transcripts had highly increased levels of PVY and consequently were more susceptible to PVY infection [64]. Recently, the study comparing laboratory isolate, transgenic line, and five wild strains of *N. benthamiana* concluded that the plant antiviral defense was compromised by its early vigor, which originated from a mutation in RDR1 rendering it inactive. It was found that laboratory strain and one wild accession from South Australia contain a 72 nucleotide insertion in their *RDR1* gene which provides survival advantage by enhancing plant early vigor. This mutation however, results in an impaired antiviral defense reflected in higher susceptibility to different families of viruses including *Potyviridae*, *Bromoviridae*, *Virgaviridae*, and *Geminiviridae*. These results confirm the vital role of RDR1 in antiviral defense [55].

### 5.3. Argonautes

siRNAs are incorporated into AGOs, which are a part of RISCs, to form the molecular platform for RNA silencing machinery. They play a vital role in plant defense mechanisms via post-transcriptional gene silencing (PTGS) or transcriptional gene silencing (TGS) of pathogen and host genes. AGO proteins are critically important for antiviral defense against RNA and some DNA viruses. AGOs encompass a large family of proteins varying among plants in number: from 4 in *Chlamydomonas reinhardtii* to 18 in *Oryza sativa* [135]. *A. thaliana* genome encodes 10 different AGOs which control the actions of different small RNAs. Several of them were observed to be involved in plant defense against different viruses including CMV, PVX, TuMV, TCV, BCTV and others (Table 1). AGOs consist of four domains: N-terminal domain, PAZ domain, MID domain, and C-terminal (PIWI) domain. It is commonly accepted that the active catalytic site of AGO proteins contains two Asp residues and one His residue (Asp–Asp–His) or three Asp residues (Asp–Asp–Asp). AGO proteins can bind virus-derived siRNA to facilitate base pairing with complementary target RNAs and the target ssRNA may be cleaved or sliced by the RNase H-like activity of AGO in the effector complexes. Therefore, AGO proteins can directly repress viral translation. Furthermore, they can indirectly affect virus replication and translation through regulation of defense genes expression. AGOs can also enhance silencing by the synthesis of cleaved RNA fragments which are substrates for RDRs. The antiviral activity of AGO proteins depends on many factors such as levels of expression, tissue and subcellular localization, AGO interacting partners, sRNA binding preferences [34]. Essential cofactors in RNA silencing reaction are proteins containing Gly-Trp (GW) dipeptides which associate with AGOs [136,137]. 

## 6. Conclusions

Plant antiviral defense is controlled by intricate interplay of biochemical, genetic, and cellular factors. In this review we described many different plant defense mechanisms involving proteins. However, it is difficult to describe every single mechanism of such kind thoroughly. We are aware that some other protein-dependent ways of plants to protect themselves against viruses also exist. For instance, antiviral responses could also be connected with RNA surveillance pathways. Nonsense-mediated decay (NMD) is a highly conserved mRNA quality control mechanism, that selectively eliminates aberrant transcripts. NMD recognizes mRNAs which prematurely terminate translation and then directs them for decay. Several viruses generate RNAs with unusual properties that are usually a consequence of their compact genome architecture. For instance, in many viruses several different proteins are encoded within a single polycistronic mRNA molecule. By recognizing viral RNAs and directing them for degradation, NMD neutralizes virus infection and functions as a basic viral restriction mechanism. In one experiment, a genetic screen in *A. thaliana* discovered that mutations in the UPF1 gene, which is a key NMD effector, enhance plant susceptibility to PVX infection [56]. It was also shown that NMD controls the expression of elements participating in plant defense including PRRs [138].

Another interesting form of plant antiviral strategy is natural recessive resistance which has been recently reviewed in great detail [139,140]. It develops when there is a mutation in a recessive gene encoding an important element for viral replication. Cellular translation initiation factors (eIF) 4E and 4G and their isoforms are examples of recessive viral resistance genes and they have been utilized in different crops against a wide range of plant viruses. Mutations in eIF4E, eIF4G and their isoforms result in loss-of-susceptibility to viruses from family *Potyviridae* and several other viruses. *A. thaliana* mutants lacking eIFiso4E, which is an isoform of eIF4E gene, were resistant to *Tobacco etch virus* (TEV) [141]. Similar eIF4E-dependent recessive resistance to potyviruses has been observed in lettuce, pepper, and wild tomato [142,143,144]. Analogous effect has been observed for other viruses including CMV, TCV in *Arabidopsis*, *Melon necrotic spot virus* (MNSV) in melon, *Bean yellow mosaic virus* (BYMV) in barley, *Rice yellow mottle virus* (RYMV) in rice, etc. [139]. Another protein involved in recessive resistance is *Arabidopsis thaliana* RNA helicase 8 (AtRH8), which has been shown to interact with viral genome-linked protein (VPg) of potyvirus and to be a key host factor for the virus infection. AtRH8-deficient mutant plants exhibited enhanced resistance to *Plum pox virus* (PPV) and TuMV with no apparent plant damage [145]. Recessive resistance mechanism relies on mutations in host disease-susceptibility genes what consequently prevents viruses from propagate in plant cells. Proteins participating in plant recessive resistance, when appropriately modified, may be potential candidates for further work on generating plants resistant to potyviruses. Genes involved in this mechanism are considered very promising targets for new antiviral approaches. 

It is important to realize that antiviral activity of plant proteins observed with the use of different strategies is not always comparable, because each of these strategies answers different questions. As a good example serve proteins which were purified and then examined in different antiviral assays. It is confirmed that such proteins are antiviral in vitro but whether or not they act in a similar way and have similar properties in their natural state in plants remains unknown. However, in vitro experiments provide some additional insights into the potential application of plant antiviral proteins. On the other hand in vivo methods such as “gene knockout technology” give us clearer views on what the antiviral function of proteins in plants is and help to identify proteins contributing to plant innate or induced antiviral defense. Nonetheless, all types of experiments need to be performed to obtain a better picture of protein function and its possible applications. For example, from practical point of view RIPs can be used to enhance plant defense against viruses and potentially other parasites. In consequence, the use of properly mutated less toxic type 1 RIPs may be less severe to transformed plants, causing less damage. Transgenic plants expressing RIPs have been exploited in agriculture to boost immunity and protect crops from viruses while minimizing the application of potentially dangerous plant protection products [60,146]. When it comes to the expression of potentially toxic proteins in plants cultivated for human consumption, researchers need to take safety issues under consideration. To our best knowledge, to date no article was published concerning the effects of edible transgenic plants expressing RIPs on humans and other animals. However, it is worth noting that the majority of endogenous RIPs are non-toxic to humans when eaten. RIPs are present in a broad range of plant species used for human consumption, such as rice, barley, tomato, maize, pumpkin, asparagus, sugar beet and even in plants which are consumed raw, e.g., spinach [92]. Additionally, thermal, chemical, and mechanical food processing usually denatures proteins in GM crops generating a loss of toxic activity. Obviously, safety assessment needs to be undertaken especially when expressing RIPs which have been reported to be toxic to humans and mammals. Furthermore, in high concentrations even non-toxic RIPs can possibly exert toxic effects. For example, some non-toxic RIPs are naturally expressed at low levels in wild-type plants, but their toxicity may be unintendedly boosted in the course of genetic modification. Another risk which should be taken into account when producing RIPs in edible plants is their allergenic potential. Risk assessment of RIP-expressing edible GM plants should be reviewed on a case-by-case basis because their potential detriment to human health is complex and depends on many factors. Scientists working on such cases may follow some science-based guidelines for the toxicological evaluation of proteins introduced into edible crops by means of agricultural biotechnology [147,148]. Similarly, RBPs can be used as antiviral agents against plant RNA viruses. Additionally toxic potential of RIPs have been exploited even in drugs against animal viruses. To make RIPs more cell selective, thus enhancing their activity, RIPs have been conjugated to antibodies, constructing immunotoxins. PAP-based immunotoxins were used in the treatment of AIDS patients and some of them (e.g., TXU (anti-CD7)-PAP) reached clinical trials where they reduced the viral burden in all HIV-1-infected adult patients without any adverse reactions. However, the dose was very low and did not suffice for sustained therapeutic levels [149]. In a dose-intensification study with GLQ223, which is a highly purified form of trichosanthin, elevated levels of CD4+ and CD8+ lymphocytes were observed in HIV-infected patients. Such effect sustained for at least 4 weeks after the last intravenous administration. The treatment was classified as safe and well tolerated by patients with mild side-effects [150]. The early phase clinical trials show the possible use of trichosanthin and PAP as possible anti-HIV agents. Phase II clinical trial involving HIV-positive patients to assess the efficacy, activity, and safety of GLQ223 have been completed [clinicaltrials.gov]. Furthermore, the development of trichosanthin-based immunotoxins, such as the one by Wang and colleagues [151], may result in enhanced protein activity and specificity.

RNA silencing-mediated approach to fight virus infections has become a powerful tool in resistant crop production. Transgenization with long hairpin RNA (hpRNA) and with artificial miRNA have been reported to provide resistance against plant RNA viruses [152,153,154]. Thorough understanding of RNA-silencing mechanisms can aid in development of new antiviral systems in plants.

Continued research on plant antiviral proteins will undoubtedly provide novel insights into their mechanisms of action and can potentially facilitate the development of new biotechnological tools to improve general crop yield and quality in agriculture. It is essential to better understand the mechanism of action of antiviral proteins to take advantage of their properties in different areas of life. These proteins may be used to develop transgenic plants that could express antiviral proteins at high levels in the presence of the pathogen, what could possibly enhance plant resistance to virus infections, reducing the loss in crops, and also decreasing the need for pesticide application. 

## Figures and Tables

**Table 1:** List of plant proteins with antiviral activity confirmed in different sets of experiments.

Protein	Family	Source Plant	Target Virus	Reference
**a.** **Proteins with antiviral properties observed in experiments involving mutant plants (Figure 1a)**				
AGO1	AGO	*Arabidopsis thaliana*	BMV	[30]
			CMV	[31,32]
			TCV	[33]
			TuMV	[34]
		*Nicotiana benthamiana*	ToRSV	[35]
		*Oryza sativa*	RSV	[36]
AGO2	AGO	*Arabidopsis thaliana*	TCV	[37,38]
			CMV	[37]
			CMV	[32]
			PVX	[39]
			TRV	[40]
			TuMV	[41,42]
		*Nicotiana benthamiana*	TBSV	[43]
AGO4	AGO	*Arabidopsis thaliana*	BCTV	[44,45]
			CMV	[46,47]
			TRV	[40]
		*Nicotiana benthamiana*	PVX	[48]
AGO5	AGO	*Arabidopsis thaliana*	PVX	[49]
			TuMV	[42]
AGO7	AGO	*Arabidopsis thaliana*	TCV	[33]
			TuMV	[42]
AGO10	AGO	*Arabidopsis thaliana*	TuMV	[42]
AGO18	AGO	*Oryza sativa*	RDV, RSV	[36]
BTR1	RBP	*Arabidopsis thaliana*	ToMV	[50]
DCL2 and DCL4 (together)	DCLs	*Arabidopsis thaliana*	PVX	[51]
			TuMV	[34]
			BMV	[30]
			TCV	[33]
DCL4	DCL	*Arabidopsis thaliana*	PVX	[51]
			TuMV	[34]
		*Nicotiana benthamiana*	PVX	[51]
DRB3	RBP	*Arabidopsis thaliana*	CaLCuV, BCTV	[45]
DRB4	RBP	*Arabidopsis thaliana*	TYMV	[52,53]
			TCV	[54]
RDR1	RDR	*Arabidopsis thaliana*	TuMV	[34]
		*Nicotiana benthamiana*	TMV	[55]
RDR6	RDR	*Arabidopsis thaliana*	TCV	[54]
			TuMV	[34]
			BMV	[30]
UPF1	Helicase	*Arabidopsis thaliana*	PVX	[56]
**b.** **Proteins with antiviral properties observed in experiments using GM plants (Figure 1b)**				
AGO2	AGO	*Arabidopsis thaliana*	CMV	[57]
			PVX	[49]
AGO5	AGO	*Arabidopsis thaliana*	CMV	[57]
			PVX	[49]
APUM5	RBP	*Arabidopsis thaliana*	CMV, TuMV	[58]
AtGRP7	RBP	*Arabidopsis thaliana*	TMV	[59]
BTR1	RBP	*Arabidopsis thaliana*	ToMV	[50]
CaPR10	PR-10	*Capsicum annuum*	TMV	[22,58]
NIK1	kinase	*Arabidopsis thaliana*	CaLCuV	[13]
PAP	RIP	*Phytolacca ameriacana*	PVY, PVX, CMV	[60]
PAP II	RIP	*Phytolacca americana*	TMV, PVX	[61]
PAP-C	RIP	*Phytolacca americana*	PVX	[62]
PIP	RIP	*Phytolacca insularis*	PVY, PVX, PLRV	[63]
PR2a	PR-2	*Nicotiana tabacum*	TMV	[23]
PR3	PR-3	*Nicotiana tabacum*	TMV	[23]
RDR1	RDR	*Nicotiana tabacum*	PVY	[64]
Trichosanthin	RIP	*Trichosanthes kirilowii*	TuMV	[65]
**c.** **Proteins exhibiting antiviral activity in vitro (Figure 1c)**				
CirA	AMP	*Chassalia parvifolia*	HIV	[66]
CirB	AMP	*Chassalia parvifolia*	HIV	[66]
Kalata B1	AMP	*Oldenlandia affinis*	HIV	[28]
Kalata B8	AMP	*Oldenlandia affinis*	HIV	[67]
Limenin	AMP	*Phaseolus limensis*	HIV-1	[29]
Lunatusin	AMP	*Phaseolus* *lunatus*	HIV-1	[68]
Phaseococcin	AMP	*Phaseolus coccineus* ‘Minor’	HIV-1	[69]
Sesquin	AMP	*Vigna sesquipedalis* cv.	HIV-1	[70]
Stellarmedin A	PR-9	*Stellaria media*	HSV-2	[21]
*unnamed*	AMP	*Phaseolus vulgaris* cv.	HIV-1	[71]

Table 1 presents antiviral proteins for which antiviral activity was detected using different experimental approaches. Section (**a**) lists proteins for which antiviral activity was observed in experiments involving mutant plants and gene knockout technology. The gene of interest was inactivated and after virus inoculation the loss-of-function mutant plants were compared with controls. Several studies employing gene silencing are also presented in this section. Section (**b**) lists antiviral proteins which were analyzed using genetically modified (GM) plants—transgenic plants or plants overexpressing the gene of interest (GOI). Briefly, the GOI from one plant was introduced to the other plant usually using *Agrobacterium*-mediated infiltration. Such GM plants were compared with controls after virus inoculation. For some proteins experiments on both mutant plants and GM plants were conducted therefore they appear in both sections of the table. Section (**c**) lists antiviral proteins which were first isolated from plants and afterwards their properties were studied in different in vitro assays. Virus name abbreviations: BCTV—*Beet curly top virus*, BMV—*Brome mosaic virus*, CaLCuV—*Cabbage leaf curl virus*, CaMV—*Cauliflower mosaic virus*, CMV—*Cucumber mosaic virus*, HIV—*Human immunodeficiency virus*, PLRV—*Potato leafroll virus*, PVX—*Potato virus X*, PVY—*Potato virus Y*, RDV—*Rice dwarf virus*, RSV—*Rice stripe virus*, TBSV—*Tomato bushy stunt virus*, TCP—*Tomato crinkle virus*, TCV—*Turnip crinkle virus*, TMV—*Tobacco mosaic virus*, ToMV—*Tomato mosaic virus*, ToRSV—*Tomato ringspot virus*, TRV—*Tobacco rattle virus*, TuMV—*Turnip mosaic virus*.

## Abbreviations

PR	pathogenesis-related proteins
RBP	RNA-binding protein
DCL	Dicer-like protein
RDR	RNA-dependent RNA polymerase
sRNA	small RNA
AGO	Argonaute protein
RISC	RNA-induced silencing complex
RIP	ribosome-inactivating protein
AMP	antimicrobial peptide
BMV	*Brome mosaic virus*
CaMV	*Cauliflower mosaic virus*
PLRV	*Potato leafroll virus*
RDV	*Rice dwarf virus*
RSV	*Rice stripe virus*
TBSV	*Tomato bushy stunt virus*
TCP	*Tomato crinkle virus*
ToRSV	*Tomato ringspot virus*
TRV	*Tobacco rattle virus*
R	resistance protein
Avr	avirulence protein
LRR	leucine-rich repeat
PRR	pattern recognition receptor
PAMP	pathogen-associated molecular pattern
HR	hypersensitive response
SAR	systemic acquired resistance
SA	salicylic acid
PAP	pokeweed antiviral protein
HSV	*Human simplex virus*
TMV	*Tobacco mosaic virus*
HIV	*Human immunodeficiency virus*
PVY	*Potato virus Y*
PVX	*Potato virus X*
CMV	*Cucumber mosaic virus*
TuMV	*Turnip mosaic virus*
RBD	RNA-binding domain
RRM	RNA-recognition motif
KH	K homology domain
ZnF	zinc finger domain
PUF	Pumilio/FBF domain
PAZ	Piwi/Argonaute/Zwille domain
APUM5	*Arabidopsis* Pumilio RNA-binding protein 5
PHD	Pumilio homology domain
AtGRP7	*Arabidopsis thaliana* glycine-rich protein 7
DRB4	dsRNA-binding protein 4
TYMV	*Turnip yellow mosaic virus*
TCV	*Turnip crinkle virus*
TLS	tRNA-like structure
CaLCuV	*Cabbage leaf curl virus*
BCTV	*Beet curly top virus*
ToMV	*Tomato mosaic virus*
siRNA	small interfering RNA
miRNA	microRNA
rasiRNA	repeat-associated siRNA
